# Incidence and predictors of tuberculosis among children on antiretroviral therapy at northeast Ethiopia comprehensive specialized hospitals, 2022; A multicenter retrospective follow-up study

**DOI:** 10.1016/j.heliyon.2022.e12001

**Published:** 2022-11-30

**Authors:** Endalk Birrie Wondifraw, Ermias Sisay Chanie, FishaAlebel Gebreeyesus, Gebeyaw Biset, Birhanu Desu Tefera, Mulusew Zeleke

**Affiliations:** a Department of Pediatric and Child Health Nursing, College of Medicine and Health Science, Wollo University, Dessie, Ethiopia; b Department of Pediatrics and Child Health Nursing, College of Health Science, Debre Tabor University, Debre Tabor, Ethiopia; c Department of Nursing, College of Medicine and Health Sciences, Wolkite University, Wolkite, Ethiopia; d Department of Emergency and Critical Care Nursing, College of Medicine and Health Science, Wollo University, Dessie, Ethiopia; e Department of Adult Health Nursing, College of Medicine and Health Science, Wollo University, Dessie, Ethiopia

**Keywords:** Incidence, Tuberculosis, Anti-retroviral therapy, Northeast, Ethiopia

## Abstract

**Introduction:**

Around the world, tuberculosis (TB) is the most common cause of mortality and morbidity in both adults and children. The incidence of tuberculosis (TB) is increased worldwide by co-infection with the human immunodeficiency virus (HIV), particularly in Sub-Saharan Africa. As a result, the study aimed to determine the incidence and predictors of tuberculosis among children on antiretroviral therapy at northeast Ethiopia Comprehensive Specialized Hospitals.

**Methods:**

An institution-based retrospective follow-up study was carried out in northeast Ethiopia’s Comprehensive Specialized Hospitals, among 362 children on antiretroviral therapy from January 1, 2007, to September 30, 2021. The data were entered into Epi Data version 4.6.1 and then exported to STATA version 16 for analysis. Bivariate and multivariable Cox proportional hazards model was used to discover tuberculosis predictors. Variables with a p-value of <0.05 at 95% confidence intervals in the multivariable Cox proportional hazard model were considered statistically significant.

**Results:**

Among the 358 Human Immunodeficiency Virus-infected children, two-thirds (69.3%) were over ten years old. The overall tuberculosis incidence rate was 2.0 (95%CI: 1.5–2.6) per 100 person-years with a total of 2452 years of observations. WHO clinical stages III and IV [AHR: 3.2 (95% CI 1.8–5.5)], being severely stunted [AHR = 2.1 (95% CI, 1.5–358)], and “Fair” and “poor” adherence levels to antiretroviral therapy [AHR = 4.0 (95% CI 1.5–10.8)] were independent predictors of tuberculosis.

**Conclusion:**

The incidence of tuberculosis in children infected with HIV/AIDS was high in this study. The risk of tuberculosis (TB) in HIV/AIDS-infected children has been linked to WHO stages III and IV, severe stunting, and "Fair" and "Poor" ART adherence. As a result, children with HIV/ADIS should be evaluated on a regular basis in order to improve the quality of ART services and reduce the incidence rate of tuberculosis among children.

## 1 Introduction

Around the world, tuberculosis (TB) is the most common cause of mortality and morbidity in both adults and children. The incidence of tuberculosis (TB) is increased worldwide by co-infection with the human immunodeficiency virus (HIV), particularly in Sub-Saharan Africa [1]. Human Immunodeficiency Virus (HIV) can induce tuberculosis (TB), which speeds up the natural development of HIV and enhances its reproduction. Tuberculosis is the most common cause of death worldwide, particularly among HIV-infected persons [1, 2, 3].

Tuberculosis remained the leading cause of death from infectious diseases in 2019. In 2019, approximately 10 million people will contract tuberculosis worldwide, with 1.2 million tuberculosis deaths among HIV-negative individuals and 208,000 among HIV-positive individuals. Adults account for 88% of tuberculosis patients, while children under the age of 15 account for 12% [4, 5].

By 2022, 40 million people with tuberculosis (TB) will be diagnosed and treated, including 3.5 million children and 1.5 million people with drug-resistant TB. In addition, 376,000 people living with HIV were diagnosed with TB, with 88% receiving life-saving antiretroviral therapy [6].

The majority of cases of HIV-associated tuberculosis (85% of TB patients have a documented HIV test result) occur in Africa, and 214,000 people living with TB were diagnosed with HIV [4, 7, 8, 9].

Tuberculosis is primarily a poor-community disease, and children with the condition usually live in impoverished areas with few healthcare options [10]. Various risk factors for mortality have been observed in African sub-regions among children with TB-HIV co-infection before or during ART; some of these children had had anti-TB treatment before ART began, while others had not [11].

Ethiopia is one of the top ten countries with the highest burden of HIV/AIDS, with an incidence rate of 341 cases per 100,000 people and HIV infecting 31% of tuberculosis patients, according to a report from the Ethiopian federal HIV and AIDS prevention and control office [12, 13]. Furthermore, pediatric tuberculosis diagnosis and treatment resources are generally limited to higher-level care facilities such as national referral hospitals and urban facilities [14, 15].

Despite advancements in treatment and prevention, the epidemiological burden of TB is not currently declining at a rate that will allow us to meet sustainable development goals. On the other hand, a global policy known as "The End TB Strategy" aims to reduce TB's incidence rate by 90% by the end of 2035 [16]. As a result, the current study has implications for ensuring this strategy's success.

Despite the fact that tuberculosis is a leading cause of hospitalization and death in HIV-infected children, there has been a general lack of prior studies on TB incidence and predictors in HIV-infected children in Ethiopia. Therefore, the study was designed to determine the incidence and predictors of tuberculosis among children on ART at northeast Ethiopia Comprehensive Specialized Hospitals.

## 2 Methods and materials

### 2.1 Study design, setting, and period

An institution-based retrospective follow-up study was conducted at northeast Ethiopia’s Comprehensive Specialized Hospitals, from January 1, 2007, to September 30, 2021. Dessie and Woldia Comprehensive Specialized Hospitals were the hospitals concerned. We chose these hospitals because they are the only comprehensive specialized hospitals in the study area with a high patient flow. The first study area was Dessie Comprehensive Specialized Hospital, which is located in Dessie Town. Dessie is located about 400 km from Addis Ababa, the capital city of Ethiopia. The hospital serves about 5.5 million people. The ART service started in 2005. From January 1, 2007, to September 30, 2021, a total of 7832 adults and 1230 children were enrolled in the ART clinic [17]. The second study area was the Woldia Comprehensive Specialized Hospital, which is located in Woldia Town, which is the capital city of the North Wollo Zone. It is located 520 km from the capital Addis Ababa. As a referral hospital, this hospital serves more than 4 million people. The ART service has been one of the services delivered since 2005. From January 1, 2007, to September 30, 2021, a total of 9325 adults and 775 pediatric patients have been enrolled in the ART clinic.

### 2.2 Study participant

All HIV-infected children under the age of 15 who began ART at northeast Ethiopia's Comprehensive Specialized Hospitals served as our source population for this study. Our study population includes all HIV-infected children under the age of 15 who were engaged in therapy between January 1, 2007, and September 30, 2021.

### 2.3 Eligibility criteria

All HIV-infected children <15 years of age who took ART for at least one month from January 1, 2007, to September 30, 2021, were eligible for this study. Children with incomplete chart recording at baseline and during the follow-up period, particularly critical information like age, sex, weight, height, ART regimen, date of ART commencement, and date of the incident, or censored reporting, were excluded from the study. We excluded four patients from this study for the reasons stated above.

### 2.4 Sample size determination and sampling procedure

**Figure 1:**
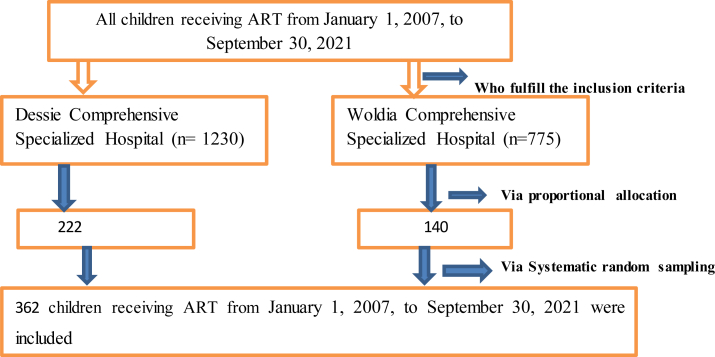
Schematic diagram of sampling procedure among children on antiretroviral therapy at northeast Ethiopia Comprehensive Specialized Hospitals.

The sample size was calculated using the Log-rank survival data analysis of the two-population proportion formula in open STATA version 16.0, with the following key assumptions: 95% confidence level, 80% power, and a 5% margin of error. It was calculated by taking significantly associated predictors from a study conducted at the Debre-Markos referral hospital in northwest Ethiopia [18]. We used WHO clinical staging as a predictor variable (WHO clinical stages III & IV as the exposed group denoted by q1 (0.25) and WHO clinical stages I & II as the control group denoted by q0 (0.35)). The total sample size was 362, accounting for 10% of incomplete medical data. The samples were distributed proportionally among the two comprehensive specialized hospitals and chosen by a computer-generated simple random sampling technique (Figure 1).

### 2.5 Variables of the study

#### 2.5.1 Dependent variable

The dependent variable for this study was the occurrence of tuberculosis infections during follow-up.

#### 2.5.2Independent variables

The independent variables were socio-demographic characteristics (e.g., age, sex, residence, and caregiver relationship), Clinical and laboratory predictors (e.g., WHO clinical stage, CD4 count or percentage, hemoglobin (Hgb) level, wasting, stunting). ART and other medication-related predictors (for example, type of baseline ART regimen, level of ART adherence, use of Isoniazid Preventive Therapy (IPT), Disclosure HIV status, and duration of follow-up in months).

### 2.6 Operational definitions

**Event**: the occurrence of tuberculosis during the follow-up time.

**Tuberculosis**: Cases were detected using sputum or stomach aspirate microscopy, chest X-ray examination, and/or histology, in accordance with the Ethiopian Ministry of Health's TB diagnosis guideline [19].

**Censored**: Lost, drop out, transfer out, died of other causes or completed study period before developing tuberculosis.

**TB-free probability time**: Consider between the start of ART and the diagnosis of tuberculosis.

**Level of ​ART adherence**: The percentage of drug dosage computed from the total monthly doses of ART drugs was used to classify the patients (Good >95%, fair 85–94%, and poor <85%) [20].

**Underweight and Stunting**: according to WHO growth curve weight/age < -3 z score and height/age < -3 z [21].

**CD4 cell count or %**: CD4 cell counts are obtained from blood work as part of laboratory monitoring for HIV infection. CD4+ cell counts are usually measured when you are diagnosed with HIV (at baseline), every 3–6 months during first 2 years or until your CD4 count increases a above 300 cells/mm^3^ [22]. Based on the child's age, CD4 below the threshold level was categorized - CD4 cell count <1500/mm3 (<25%) for <12 months, CD4 cell count <750/mm3 (<20%) for age 12–35 months, CD4 cell count <350/mm3 (<15%) for age 36–59 months, and CD4 cell count <200/mm3 (<15%) for age ≥60 months [23].

**Disclosure of HIV status**: When children are told the name of the illness (HIV and/or AIDS disease-specific information) and how they acquired the disease [24].

### 2.7 Data collection tools and procedures

The data extraction tool was created using the patient register book from the Ethiopian Federal Ministry of Health. From 1 January 2007 to 30 September 2021, data was collected using ART patients' record cards and registers. The data extraction tool included information on socio-demographics, clinical care, and treatment. Four BSc nurses collected the data under the supervision of one MSc nursing practitioner. A one-day training session on data collection and monitoring processes was held.

### 2.8 Data quality assurance

A pre-test was performed on 5% of the sample size of medical records at Dessie Compressive Specialized Hospital to ensure that they were complete. To avoid duplication, data extracted from patient records were coded. The data collectors, supervisor, and principal investigator double-checked the completed formats for accuracy daily, and cleaning was done during the data collection and analysis process.

### 2.9 Data processing and analysis

Before being transferred to STATA version 16 for analysis, the data was entered into EPI Data version 4.6.1. Tables and graphs were used to explore the descriptive statistics. By dividing the number of children who developed TB throughout the follow-up period by the number of years, the incidence rate was computed. The Kaplan-Meier curve was used to determine the survival time. Additionally, the log-rank test was used to evaluate the curve difference between the predictor variables. Schoenfeld residual ph test and Log ph plot were used to verify the Cox-proportional hazard regression model's necessary assumptions. For each predictor variable, bivariate cox-proportional hazard models were applied. A multivariate cox-proportional hazard model was fitted with components that had a bivariate p-value of less than 0.25, and an adjusted hazard ratio (AHR) with a 95% confidence interval (CI) was calculated. Finally, factors with a P-value of ≤0.05 were considered statistically significant.

#### 2.9.1 Ethical approval and consent to participate

Ethical clearance was obtained from Wollo University, College of Medicine and Health Science, department of pediatrics, and child health nursing ethical review committee. The reference number for this letter was PCHN-250/2022. Each hospital administrator was also given a permission letter. Our study used secondary data, so we did not need consent from the patient. In order to maintain anonymity, the names and other identifiers of study participants were not included. This study was conducted following the signing of the Helsinki Declaration.

## 3 Result

### 3.1 Socio-demographic characteristics

A total of 362 HIV-infected children were enrolled in ART during the follow-up period. Four were excluded due to incomplete data. Two-thirds (69.3%) of the 358 HIV-infected children were over ten years old. More than half of the 193 participants (53.9 %) were men. The majority of the children, 298 (83.2 %), lived in cities, and two-thirds of the caregivers, 233 (65.1 %), were married (Table 1).

**Table 1:** Socio-demographic characteristics of children on antiretroviral therapy at northeast Ethiopia Comprehensive Specialized Hospitals, 2022.

Characteristics		Frequency (n = 358)	Percentage
Age of the chid (years)	<5 years	65	18.2
	5–9 years	45	12.6
	≥10 years	248	69.3
Sex	Male	193	53.9
	Female	165	46.1
Residence	Urban	298	83.2
	Rural	60	16.8
Relation of the caregiver to the child	Parent	317	88.5
	Sister/brother	22	6.1
	Uncle/aunt	8	2.2
	Grandparent	11	3.1
Marital status of caregiver	Single	12	3.4
	Married	233	65.1
	Divorced	14	3.9
	Widowed	99	27.7
Caregiver’s occupation status	House wife	207	57.8
	Governmental employee	81	22.6
	Non-governmental employee	29	8.1
	Merchant	21	5.9
	Farmer	20	5.5

### 3.2 Clinical and treatment-related characteristics

Out of 358 HIV-infected children, 284 (79.3%) developed drug-side effects. Of the total children, 40 (11.2%) developed treatment failure. Two-thirds of 221 (61.7%) children's CD4 counts or % level was below the threshold and 146 (40.8%) developed WHO stage III and IV. Nearly half of the 358 HIV-infected children (58.7%) were receiving Cotrimoxazole prophylactic therapy (CPT), while 101 (28.2%) were receiving isoniazid prophylactic therapy (IPT). Two-thirds of 254 (70.9%) children had a good level of adherence to ART during the follow-up period. The majority of children 336 (93.9%) had Hgb levels <10 mg/dl. On the other hand, almost half of the children, 188 (52.5%), HIV-infected children, developed an opportunistic infection during the follow-up period. Among these children, 16.7% develop tuberculosis, 13.1% develop bacterial pneumonia, 12.3% develop herpes zoster and 10.4% develop other opportunistic infections. Out of 358 HIV-infected children, 140 (39%) were treatment started with an EFV regimen, and 321 (89.7%) were followed for more than 34 months (Table 2).

**Table 2:** Clinical and treatment-related characteristics of children on antiretroviral therapy at northeast Ethiopia Comprehensive Specialized Hospitals, 2022.

Characteristics		Frequency (n = 358)	Percentage
Drug side effect	Yes	284	79.3
	No	74	20.7
Treatment failure	Yes	40	11.2
	No	318	88.8
CD4 counts or % level	Below threshold	221	61.7
	Above threshold	137	38.3
WHO clinical staging	I/II	212	59.2
	III/IV	146	40.8
IP	Given	101	28.2
	Not given	257	71.8
CPT	Given	210	58.7
	Not given	148	41.3
Weight for age	Normal	308	86
	Underweight	50	14
Height for age	Normal	314	87.7
	Stunting	44	12.3
Adherence	Good	254	70.9
	Fair/Poor	104	29.1
Duration of follow-up in months	<34 months	37	10.3
	>34 months	321	89.7
Opportunistic infections	Yes	188	52.5
	NO	170	47.5
Levels of HGB	>10 mg/dl	22	6.1
	≤10 mg/dl	336	93.9
Initiation regimen	EFV based	140	39
	NVP, PI and other based	218	60.1

### 3.3 Tuberculosis incidence rate during follow-up

The overall tuberculosis incidence rate among HIV-infected children was 2.0 (95% CI: 1.5–2.6) per 100 person-years. The children followed with a range from 04 to 156 months, which yields a total of 29,424 months or 2452 years at risk.

### 3.4 Predictors of TB incidence in HIV positive children on ART

In the bivariate Cox proportional hazard model, age, sex, residence, the caregiver's relationship with the child, initial ART regimen, duration of follow-up in months, IPT, CPT, CD4 counts or % level, Hgb level, WHO stage, weight for age, height for age, disclosure of HIV status, and level of adherence to ART of variables had a P-value less than or equal to 0.25 and entered into for multivariate cox proportional hazard.

In the multivariate Cox proportional hazard model, WHO clinical stages III and IV, severe stunting, and "Fair" and "Poor" adherence levels were significant predictors of tuberculosis among HIV-infected children.

The hazards of tuberculosis infection in children with WHO clinical stages III and IV were 3.2 times higher than those in children with WHO clinical stages I and II [AHR = 3.2 (95% CI 1.8–5.5)].

Being severely stunted increased the hazard of tuberculosis infection by 2.1 [AHR = 2.1 (95% CI, 1.5–3.5)] times compared to children who were not stunted.

**Figure 2:**
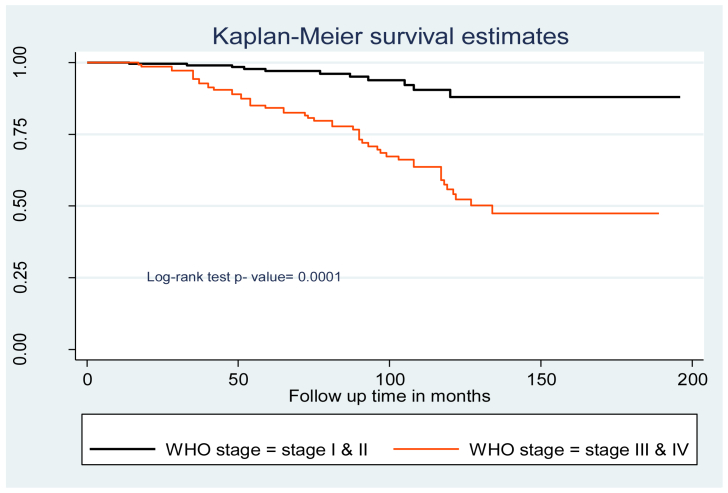
Kaplan Meier survival curve of WHO stage among children on antiretroviral therapy at northeast Ethiopia Comprehensive Specialized Hospitals, 2022.

**Figure 3:**
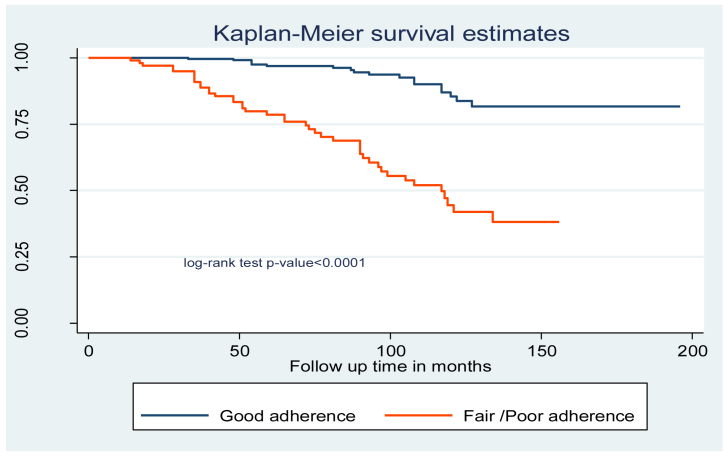
Kaplan Meier survival curve of ART adherence level among children on antiretroviral therapy at northeast Ethiopia Comprehensive Specialized Hospitals, 2022.

**Figure 4:**
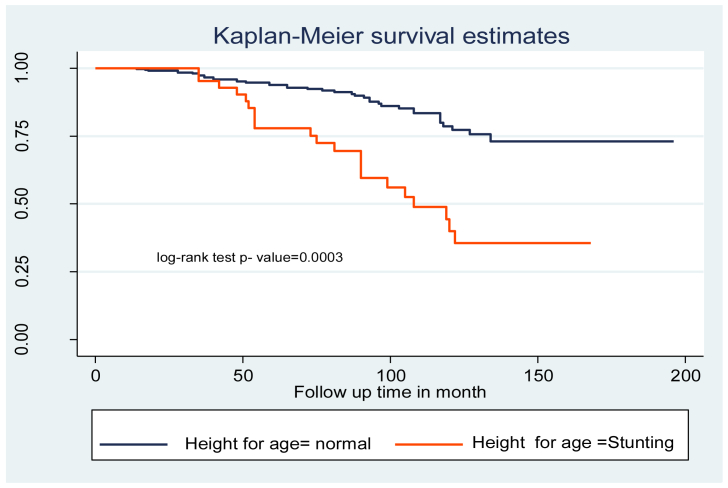
Kaplan Meier survival curve of height for age among children on antiretroviral therapy at northeast Ethiopia Comprehensive Specialized Hospitals, 2022.

“Fair” and “poor” adherence levels to ART were 4 times [AHR = 4.0 (95% CI 1.5–10.8)] more at risk of developing tuberculosis infection compared to “good” ART adherence (Table 3). In addition, the log-rank test of the predictors' between-category variables was calculated (Figures 2, 3, and 4).

**Table 3:** Cox-proportional hazard analysis of ​predictors of ​TB incidence among children on antiretroviral therapy at northeast Ethiopia Comprehensive Specialized Hospitals, 2022.

Variables		Survival status		CHR (95% CI)	AHR (95% CI)
		Event	Censored		
Age of the child (years)	<5 years	10	55	0.85(0.43–1.70)	0.99(0.43–2.27)
	5–9 years	7	38	0.95(0.43–2.13)	1.83(0.72–4.60)
	≥10 years	43	205	1	1
Sex	Male	37	156	0.62(0.37–1.05)	0.58(0.33–1.04)
	Female	23	142	1	1
Residence	Urban	44	254	2.42(1.36–4.31)	1.78(0.89–3.52)
	Rural	16	44	1	1
Relation of the caregiver to the child	Parent	52	265	0.68(0.16–2.82)	0.99(0.15–6.35)
	Sister/brother	4	18	0.88(0.16–4.84)	0.67(0.07–5.94)
	Uncle/aunt	2	6	0.75(0.10–5.34)	0.77(0.73–8.25)
	Grandparent	2	9	1	1
CD4 count or %	Below threshold	17	204	1	1
	Above threshold	43	94	0.4(0.29–0.55)	0.82(0.53–1.03)
WHO clinical staging	I/II	11	201	1	1
	III/IV	49	97	5.6(2.92–10.83)	3.23(1.88–5.55)∗∗
IP	Given	19	82	1	1
	Not given	41	216	0.99(0.57–1.60)	0.92(0.50–1.78)
CPT	Given	35	175	1	1
	Not given	25	123	0.96(0.57–1.60)	1.23(0.68–2.24)
Levels of HGB	>10 mg/dl	7	15	0.26(0.12–0.59)	0.34(0.11–8.23)
	≤10 mg/dl	53	283	1	1
Weight for age	Normal	44	264	1	1
	Underweight	16	34	2.08()1.17–3.70)	1.02(0.49–2.12)
Height for age	Normal	39	275	1	1
	Stunting	21	23	3.45(2.03–5.87)	2.14(1.56–3.59)∗
Disclosure status	Disclosed	27	155	1	1
	Non disclosed	33	143	1.46(0.87–2.43)	1.05(0.56–1.96)
Adherence	Good	18	236	1	1
	Fair/Poor	42	62	2.47(2.07–3.63)	2.05(1.16–2.37)∗∗
Duration of follow-up in months	<34 months	8	29	0.15(0.07–0.32)	1.46(0.52–4.12)
	>34 months	52	239	1	1
Initiation regimen	EFV based	27	113	0.70(0.42–1.17)	0.64(0.35–1.18)
	NVP, PI and other based	33	185	1	1

**Notice** - ∗Significant at <0.05 ∗∗ Significant at <0.01; CHR: Crude hazard ratio; AHR: adjusted hazard ratio; 1: reference category; CI: confidence interval CPT: cotrimoxazole prophylactic therapy; IPT: isoniazid prophylactic therapy.

## 4 Discussion

This study determined that the overall incidence rate of tuberculosis among children receiving ART in the comprehensive specialized hospitals in northeast Ethiopia was 2.0 (95% CI: 1.5–2.6) per 100 person-years. This result was similar to that of research conducted in Ethiopia's northwest [18] and SNNPR regions [25] and in South Africa [26]. It could be due to shared socioeconomic and demographic factors, HIV treatment facilities, and HIV treatment recommendations.

However, the incidence rate of tuberculosis in this study was higher than in previous studies undertaken in Latin America [27], the UK and Ireland [28], Europe and North America [29], and China [30]. This discrepancy could be explained by changes in the study period and setting. In poorer countries, the technique of providing treatment is typically substandard and varies widely. Furthermore, poverty, overcrowding, having a large family, and living in poor conditions may all contribute to an increased tuberculosis incidence rate in Ethiopia [31, 32].

This study found that the hazards of tuberculosis infection in children with WHO clinical stages III and IV were 3.2 times higher than those in children with WHO clinical stages I and II. This finding is supported by studies conducted in northwest Ethiopia [18, 33], Tanzania [34], South Africa [35], Pakistan [36], and China [30]. WHO clinical stages III and IV have a major impact on the risk of immunological weakening in HIV/AIDS patients, which leads to a worsening prognosis. In addition, immunity loss in advanced WHO clinical stages hastens the transition from latent to active tuberculosis infection. At this age, children should have routine examinations [18, 37].

This study also revealed that being severely stunted increased the hazard of tuberculosis infection by 2.1 times compared to children who were not stunted. Similar studies have been conducted in hospitals in the northwest, Ethiopia [38], Tanzania [39], and Uganda and Zimbabwe hospitals [40]. It's possible that HIV infection causes increased nutrient malabsorption as a result of metabolic changes, resulting in weight loss and stunting over time and exposing people to opportunistic infections earlier. Rapid viral replication, on the other hand, depletes body energy and creates an ideal environment for the emergence of lethal opportunistic infections [41].

According to this study, "fair" and "poor" adherence to ART were four times more likely to develop tuberculosis infection than "good" adherence. These findings are supported by research conducted in northwest Ethiopia [18]. Adherence to "fair" or "poor" ART offers a favorable environment for viral replication and may hasten the development of ART resistance, which can lead to an increase in viral load, a loss in immune function, and the spread of opportunistic infections [18, 42].

### 4.1 Strength of the study

This study is a long-term follow-up study that can provide a robust estimate of the incidence and predictors of tuberculosis among children on antiretroviral therapy at northeast Ethiopia Comprehensive Specialized Hospitals.

### 4.2 Limitations of the study

One of the study's limitations is its retrospective nature. As a result, clinically relevant predictor variables such as children's educational status and family economic status, as well as community hygiene practices and patients' and caregivers' awareness levels were omitted from this study.

## 5 Conclusion

In this study, the incidence rate of tuberculosis in HIV-infected children was found to be high. The risk of TB incidence rate in HIV/AIDS-infected children was associated with the WHO clinic stages III and IV, being severely stunted and “Fair” and “poor” adherence levels to ART. As a result, children with HIV/ADIS should be constantly evaluated to improve their nutritional status and level of adherence, as well as clinical stages, to improve the quality of ART services and reduce the incidence rate of tuberculosis among children.

Declarations.

### Author contribution statement

Endalk Birrie Wondifraw, Ermias Sisay Chanie: Conceived and designed the experiments; Performed the experiments; Analyzed and interpreted the data; Wrote the paper.

Fisha Alebel Gebreeyesus: Conceived and designed the experiments; Wrote the paper.

Gebeyaw Biset, Birhanu Desu Tefera: Analyzed and interpreted the data; Wrote the paper.

Mulusew Zeleke: Performed the experiments; Analyzed and interpreted the data; Wrote the paper.

### Funding statement

This research did not receive any specific grant from funding agencies in the public, commercial, or not-for-profit sectors.

### Data availability statement

Data will be made available on request.

### Declaration of interest’s statement

The authors declare no conflict of interest.

### Additional information

No additional information is available for this paper.
